# Cognitive-behaviour therapy for patients with Abridged Somatization Disorder (SSI 4,6) in primary care: a randomized, controlled study

**DOI:** 10.1186/1471-244X-8-47

**Published:** 2008-06-22

**Authors:** Rosa Magallón, Margalida Gili, Sergio Moreno, Natalia Bauzá, Javier García-Campayo, Miquel Roca, Yolanda Ruiz, Eva Andrés

**Affiliations:** 1 Department of Family Medicine, Arrabal Health Centre and University of Zaragoza, Spain; Grupo Aragonés de Investigación en Atención Primaria, Red de Actividades Preventivas y de Promoción de la Salud (REDIAPP) (G06/018), Instituto Aragonés de Ciencias de la Salud (IACS), Spain; 2 Departament of Psychology. Illes Balears University, Palma de Mallorca, Spain; 3 Department of Psychiatry, Miguel Servet Hospital and University of Zaragoza, Spain; 4 Unit of Psychiatry and Clinic Psychology, Juan March Hospital, Illes Balears University, Palma de Mallorca, Spain

## Abstract

**Background:**

Somatoform disorders are characterized by the presence of multiple somatic symptoms without an organic cause that completely explains their symptoms.

These patients generate a high cost in health services. We aim to evaluate the effectiveness and feasibility of a cognitive-behaviour therapy (CBT) programme, administered in group and individual formats in primary care for patients who are diagnosed with abridged somatization disorder.

**Method/design:**

*Design: *Multicentre, randomized, controlled trial involving 3 groups, one of which is the control group consisting of standardized recommended treatment for somatization disorder in primary care (Smith's norms) and the 2 others, the intervention groups, consisting of cognitive-behavioural therapy (10 sessions) administered in individual format (intervention group 1) or in group format (intervention group 2).

*Setting: *29 primary care health centres in the province of Zaragoza and 3 primary care health centres in the province of Mallorca, Spain.

*Sample: *N = 204 patients, (68 in each of the three groups), aged 18–65 years, able to understand and read Spanish, who fulfil Escobar's criteria of Abridgged Somatization Disorder (SSI 4,6), stable with pharmacotherapy over the previous month, and who will remain stable for the next 3 months in the doctor's opinion, having signed informed consent.

*Intervention: *Control group: Standardized recommended treatment for somatization disorder in primary care (Smith's norms). Intervention group: 10 weekly sessions of CBT, following a protocol designed by Prof. Escobar's group at UMDNJ, USA. There are 2 different treatment conditions: individual and group format.

*Measurements: *Survey on the use of health services, number and severity of somatic symptoms, anxiety, depression, quality of life and clinical global impression. The interviewers will not know which group the patient belongs to (blind). The assessments will be carried out at baseline, post-treatment, 6 months and 12 post-treatment.

*Main variables: *Utilization of health services, number and severity of somatic symptoms.

Analysis: The analysis will be per intent to treat. We will use the general linear models of the SPSS v.15 statistical package, to analyse the effect of treatment on the result variable (utilization of health services, number and severity of somatic symptoms).

**Discussion:**

It is necessary to develop more effective psychological treatments for somatoform disorders. This randomised clinical trial will determine whether cognitive behaviour therapy, both in group or in individual format, is effective for the treatment of these patients.

**Trial registration:**

Current controlled trials ISRCTN69944771

## Background

Somatoform disorders, according to the Diagnostic and Statistical Manual of Mental Disorders [1] are characterized by the presence of multiple somatic symptoms without an organic cause that completely explains their symptoms. This group of disorders include functional somatic syndromes such as fibromyalgia, chronic fatigue syndrome or irritable bowel syndrome, diseases increasingly diagnosed at present [2-4]. The most extreme form of this group is somatization disorder, a chronic and polysimtomatic disorder, which is characterized by at least four unexplained symptoms (two gastrointestinal, one sexual and one pseudoneurological symptoms) [1].

Epidemiological research studies [4,5] demonstrate that somatoform disorders show a prevalence of 0.1–0.2% in the general population and 5% in primary care settings. Although there are no specific studies on the prevalence of undifferentiated somatoform disorder (less restrictive in their diagnostic criteria), it is considered the most common disorder of the group, with a prevalence rate of up to 4.4% in the general population [6] and 22% in primary care settings [7]. In the United States it is estimated that health costs of this group of diseases accounts for 10% of the country's total health care costs [8]. The high percentage of work incapacity of these patients, the use of social services and high psychiatric comorbidity suggest the need to develop systematic research on these disorders [9]. In recent years, an abridged form of somatization disorder, more prevalent than and with the same severity of complications and use of health services as somatization disorder, has been described for research purposes [9].

Somatizing patients mostly attend their primary care physician [7], so a set of recommendations, called "Smith's norms" has been developed for the care of these patients in primary care settings [10,11]. These norms include regular appointments, systematic exploration of the symptoms referred to by the patient, avoidance of unnecessary diagnostic tests and referrals to appropriate mental health specialists, among others. At the pharmacological level, research on specific somatoform disorders are rare, so there is no a solid foundation on which to build reliable pharmacological recommendations [12]. However, studies do exist on the pharmacological treatment of pain (the main symptom of most somatoform disorders). According to these studies, antidepressant drugs (tricyclic antidepressants, mirtazapine, venlafaxine and duloxetine) [13,14] achieved a significative improvement in these disorders. For the psychological treatment, several intervention programmes (both in individual and group formats) have been developed. They are based on psychodynamic psychotherapy [15-17], educational interventions [10,11] and cognitive-behavioural psychotherapy [18-20]. However, there are few studies that evaluate the effectiveness of these interventions [14].

## Methods/design

### Objectives

The general aim is to evaluate the effectiveness and applicability of a programme of cognitive-behavioural therapy (individual and group formats) for Abridged somatization disorder (SSI 4,6) [9] compared with usual treatment.

The specific aims are:

To determine the effectiveness and cost-effectiveness of group compared with individual cognitive-behavioural intervention for patients with Abridged somatization disorder.

To identify the variables that predict greater effectiveness of cognitive-behavioural therapy in somatizing patients.

To determine whether gender differences exist for the variables included in the study.

To assess the influence of work incapacity in the evolution of the symptoms.

### Design

**Figure 1:**
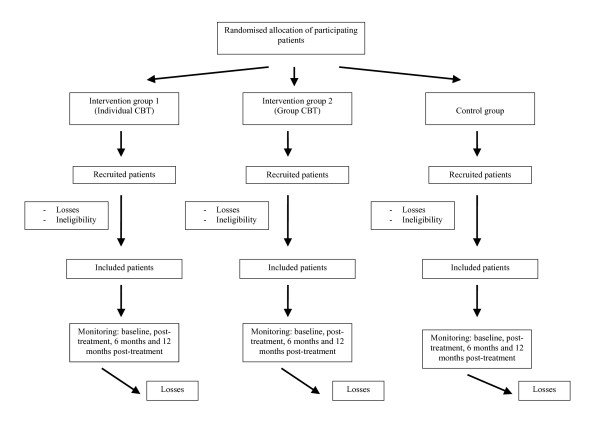


This is a multicentre, controlled trial with a random allocation of patients into three alternative branches (see Figure 1):

1. Standard treatment or "Smith's norms" (control group)

2. Individual cognitive-behavioural therapy (CBT) (intervention group 1)

3. Group CBT (intervention group 2).

The evaluation of the treatment outcomes will be performed at patient level and they will be assessed individually.

### Setting and study sample

Patients will be recruited from any of the 29 primary health care centres in the province of Zaragoza and 3 in the province of Mallorca, Spain. Patients will be recruited by doctors working in these primary care centres until the required sample is completed, without a quota of patients assigned for each centre.

Patients considered for **inclusion **are those aged 18–65 years, able to understand and read Spanish, who fulfil Escobar's criteria of Abridged Somatization Disorder (SSI 4,6) [9], stable with pharmacotherapy over the previous month, who will remain stable for the next 3 months in the doctor's opinion, and who have signed informed consent. Those **excluded **will be patients with any primary psychiatric diagnosis other than somatization disorder, severe personality disorder that prevents an adequate implementation of the protocol for evaluation and/or intervention, inability to attend intervention sessions and refusal to participate.

### Randomization, allocation and masking of study groups

Family doctors from the health centres involved in the study who suspect a patient might fulfil abridged somatization disorder criteria [9] will administer a screening interview to determine whether the patient meets the inclusion criteria. Patients who supposedly fulfil these criteria will be interviewed, within the next 10 days, by a member of the research team for diagnostic confirmation (baseline). Cases are considered of patients who are diagnosed with abridged somatization disorder [9] using the Standardized polyvalent psychiatric interview or SPPI [21].

They will be randomly assigned to the three experimental conditions: individual cognitive-behavioural therapy, group cognitive-behavioral therapy or usual treatment in primary care. Each patient will be allocated to one of both intervention groups or to the control group by means of a computer-generated random number sequence. The allocation will be carried out by an independent person, belonging to REDIAPP (Research network on preventative activities and health promotion), who is not involved in the study. The method used to implement the random allocation sequence will be a central telephone. The sequence will be concealed until interventions are assigned. Patients agree to participate before the random allocation and without knowing which treatment they will be allocated to. CBT treatment will be administered by two psychologist (SM, NB). Study personnel conducting psychological intervention (SM, NB) and assessments (YR, JGC, RM, MR, MG) will be masked to participants' treatment conditions.

### Intervention

Control group or standardized recommended treatment for somatization disorder in primary care (Smith's norms) [10,11]: standardized letter to the family doctor with Smith's norms that includes: 1. Provide brief, regularly scheduled visits. 2. Establish a strong patient-physician relationship. 3. Perform a physical examination of the area of the body where the symptom arises. 4. Search for signs of disease instead of relying of symptoms. 5. Avoid diagnostic tests and laboratory or surgical procedures. 6. Gradually move the patient to being "referral ready".

Experimental or intervention group: establishment of the protocol developed by Prof. Escobar et al [22] from the University of New Jersey that includes 10 weekly sessions of treatment and evaluation over 4 designated times: Time 1 (baseline), Time 2 (aftercare), Time 3 (6 months post-intervention), and Time 4 (1 year post-intervention).

There will be two different treatment conditions following the same protocol: individual and group formats. Inclusion in any of the 3 conditions will be random.

### Measurements

The study personnel that carried out the measurements (JGC, RM, YR, MG) will be unaware of which treatment the patients is being allocated ("blind"). The follow-up assessments will take place at 4 designated times: time 1 (baseline), time 2 (aftercare), time 3 (6 months post-intervention), and time 4 (1 year post-intervention).

Variables and instruments of measurements (see Table 1)

**Table 1:** Study variables

Instrument	Assessment are^a^	Applied by	Times(s) of assessments
Sampling form	Age, sex, inclusion/exclusion criteri^a^	Family doctor	Screening
Othmer and Souza Screening	Somatoform disorder	Family doctor	Screening
Socio-demographic data form	Age, sex, marital status, educational level, socio-economic group, occupation	Research assistant	Baseline
EPEP psychiatric interview	Psychiatric diagnosis	Research psychiatrist	Baseline
SOMS	Somatic symptoms	Research assistant	Baseline
SSQ	Somatic symptoms	Research assistant	Baseline
SSS	Somatic symptoms	Research assistant	Baseline and all follow up sessions*
Self-declared health services	Use of health services	Research assistant	Baseline and all follow up sessions*
HAM-D	Clinical depression	Research assistant	Baseline and all follow up sessions*
HAM-^a^	Clinical Anxiety	Research assistant	Baseline and all follow up sessions*
SF-36	Health and quality of life	Research assistant	Baseline and all follow up sessions*
CGI	Clinical impression	Research assistant	Baseline and all follow up sessions*

*All follow-up sessions: baseline, post-treatment, 6 months and 12 months post-treatment

Main outcome variables:

- SSS (Severity of somatic symptoms scale) [22]: a scale of 40 somatic symptoms assessed by a 7-point visual analogue scale;

- SSQ (Somatic symptoms questionnaire) [22]: a scale made up of 40 items on somatic symptoms and patients' illness behaviour,

- SOMS (Screening for somatoform symptoms) [23,24]: a 53-item screening questionnaire for somatoform disorders with a threshold of 7 symptoms.

- Utilization of healthcare services. Survey of the treatments received (types of drug treatments, individual or group psychological treatment, use of alternative medicines, etc.).

Secondary variables:

- Socio-demographic variables: age, gender, marital status, level of education, income, professional qualification, occupation, current employment status. Survey for the family doctor on clinical characteristics of the patient (to be completed by the interviewer based on the medical record)

- SPPI (Standardized polyvalent psychiatic interview) [21]: Psychiatric interview designed by our group to diagnose psychiatric morbidity in primary care settings [4], that permits DSM-IV and ICD-10 psychiatric diagnosis. If the overall score of any of these sections is equal to or greater than 2, it is considered a positive score in the SPPI and therefore a "psychiatric case".

- Diagnosis of psychiatric or somatic disease. Causal attribution of the symptoms (psychiatric or physical attribution) according to the doctor.

- Anxiety and depression levels assessed by Hamilton Test for Anxiety and Depression (HAM-A and HAM-D) [25,26].

-Clinical global impression (CGI).

- Quality of life assessed by SF-36 (Survey of health and quality of life) [27]: Instrument that explores quality of life related to physical and mental health.

### Statistical methods

#### Sample size

To calculate the sample size we consider that the effectiveness of usual treatment (Smith's norms) is rather low, estimated at about 20% in most of the variables [10,11]. We aim to assess whether the new intervention is at least 20% more effective than usual treatment. Assuming an alpha risk of 0.05 and a beta risk of <0.20 in a bilateral contrast, we would need a sample size of 62 patients with abridged somatization disorder in each group [28]. Furthermore, if we expect a 10% loss, ie, people who do not complete the study period, the necessary sample size would be 68 patients in each group, i.e. a total sample of 204 patients.

#### Analysis strategy

Analysis will be per intent to treat. First we will compare the two intervention groups (individual and group cognitive-behaviour therapy) and the control group in order to verify that there are no significant differences among them (socio-demographic characteristics, clinical baseline data, etc). We will use the mean (standard deviation) in the continuous variables and percentages in the categorical variables. For comparisons we will use the Kruskal-Wallis test for continuous variables and the Chi-squared test for categorical variables. Non-parametric tests may also be used.

The main variables of the result are SSS, SSQ, SOMS and utilization of healthcare services at 1-year. Process variables include severity of depression (HAM-D) and anxiety (HAM-A), clinical global impression (CGI), quality of life (SF-36), and socio-demographic variables.

We will use the general linear models of the SPSS 15 statistical package, to analyse the effect of the treatment on the continuous result variables (SSS, SSQ and SOMS). We will use the analyses of linear mixed models to analyse the effect of the continuous process variables (depression, anxiety, clinical impression, quality of life).

### Ethical aspects

Informed consent will be obtained from the participants before they are aware of which group they are to be included in. Before they give their consent, the patients will be provided with a general overview of the aims and characteristics of the study and the different interventions. They will also be informed that they will be participating voluntarily, and that they can choose to withdraw at any time with the guarantee that they will continue to receive the treatment considered most appropriate by their doctor. The family doctor of any patients considered "psychiatric cases" after administering the psychiatric interview will be informed recommending appropriate treatment as usual.

The study follows Helsinki convention norms and posterior modifications and the Declaration of Madrid of the World Psychiatric Association. The study protocol was approved by the Ethical review board of the regional health authority in February 2006 (ref: FIS PI05/2185).

### Forecast execution dates

Initial recruitment of patients: June 2008

Finalisation of patient recruitment: December 2008

Finalisation of patient monitoring period: December 2009

Publication of results: March 2010

## Discussion

The cognitive-behavioural model emphasises the interaction between physiological aspects, cognitions, feelings, behaviour and environment. From this perspective, somatoform disorders show distortions at three levels [28,29]: cognitions (attention focused on the functioning of the body with a somatic attribution of the symptoms), behaviour (physical inactivity and continuous seeking of medical care) and emotions (anxiety and depression). Furthermore, physiological arousal produces a tendency to amplify somatosensory information (patients are more sensitive to bodily sensations). The use of cognitive-behavioural therapy in somatoform disorders has proven its effectiveness in several controlled studies, both in group and individual formats (14–20, 29–31). However, studies with larger samples and longer follow-up are necessary to confirm that lasting improvements are achieved.

The project presented is a replication of the study carried out by Prof. Escobar [22] in the USA which has proven effective in the treatment of somatizing patients in primary care. The specific features of this project are the following:

- Originality: This is the first intervention study on somatoform disorders in Spain and one of the first at the international level that intends to conduct psychological intervention study in a large sample (N = 204) of primary care patients with somatoform disorders. In addition, up to now there have been no studies comparing cognitive-behavioural therapy administered in groups vs. individual.

- Clinical impact: It is estimated that more than 20% of primary care consultations are due to symptoms of unknown etiology associated with stress and other psychosocial variables.

- Plannification of health resources: Developing effective treatment protocols allows better planning of human and technical resources as well as training of health professionals on the diagnosis and treatment of these disorders [32].

The main limitations and problems expected are refusals to participate in the study (due to the negative attitude of these patients towards psychiatric treatments) or change in employment status or incapacity to work over the time. To address the possible bias of disability, which act as a confounding variable in the analysis of data throughout the project will evaluate the employment status of patients who participate in the intervention, asking at each meeting whether it had changed their status (end of absence from work, disability... trial). This will attempt to statistically control the influence of this variable in the analysis of statistical data.

## Competing interests

The authors declare that they have no competing interests.

## Authors' contributions

RM, MR, JG–C and MG are the principal researchers and developed the original idea for the study and the study design. The other members of the team also participated in the design and planning of the intervention that is evaluated here. EA developed the statistical methods. All authors have read and corrected draft versions, and approved the final version.

## Pre-publication history

The pre-publication history for this paper can be accessed here:


